# A Mild Neurodevelopmental Phenotype Associated with a Novel *BRAT1* Variant: Expanding Genotype–Phenotype Correlations

**DOI:** 10.3390/medsci14060641

**Published:** 2026-10-06

**Authors:** Camilla Meossi, Lorenzo Cipriano, Flavio Dal Canto, Stefano Pagano, Giulia Nutile, Alessandra Tessa, Carla Cona, Gianmarco Dalla Zanna, Roberta Battini, Domenico Montanaro, Filippo M. Santorelli

**Affiliations:** 1 Department of Developmental Neuroscience, IRCCS Stella Maris Foundation, Via dei Giacinti 2, 56128 Pisa, Italystefanop@hotmail.it (S.P.);; 2 Molecular Medicine, IRCCS Fondazione Stella Maris, 56128 Pisa, Italy; 3 Department of Clinical and Experimental Medicine, University of Pisa, 56126 Pisa, Italy; 4 U.O.S. Dipartimentale e Servizio Autonomo di Risonanza Magnetica, IRCCS Stella Maris Foundation, 56128 Pisa, Italy

**Keywords:** Western blot, *BRAT1* disease spectrum, neurodevelopmental disorders, cerebellar hypoplasia, gait ataxia, skin punch biopsy

## Abstract

**Background:** *BRAT1*-related disorders constitute a continuous neurodevelopmental spectrum ranging from rigidity and multifocal seizure syndrome, lethal neonatal form (RMFSL) to milder phenotypes characterized by cerebellar involvement and variable developmental impairment. Genotype–phenotype correlations remain incompletely defined, particularly for missense variants that may retain residual function. **Objectives:** To characterize clinical and molecular findings in a patient with a mild *BRAT1*-related phenotype. **Methods:** An 18-year-old boy underwent clinical, neuroradiological, neurophysiological, and genetic evaluations. Exome sequencing with in silico filtering on an ataxia gene panel and parental segregation analysis was performed. BRAT1 protein expression was assessed by Western blot analysis in patient-derived fibroblasts. **Results:** We report an 18-year-old male carrying compound heterozygous *BRAT1* variants, including a recurrent truncating variant (c.294dup) and a novel missense variant (c.1784A>T; p.(Glu595Val)). The patient presented with early-onset global developmental delay, cerebellar ataxia, dysarthria, action tremor, and oculomotor abnormalities without epilepsy. Brain MRI showed marked hypoplasia of the inferior cerebellar vermis associated with a mega cisterna magna, without involvement of the cerebellar hemispheres. Follow-up over more than fifteen years showed relatively preserved functional autonomy in late adolescence (SARA 8.5/40; FARS-ADL 11/36). Western blot analysis of patient-derived fibroblasts revealed low but detectable BRAT1 protein levels (approximately 15–20% of mean control expression), indicating reduced but retained BRAT1 protein expression, compatible with, but not proof of, a residual-function effect. **Conclusions:** These findings are compatible with a contribution of residual BRAT1 protein expression to phenotypic attenuation in this patient and further expand the mild end of the BRAT1-related disease spectrum. Functional assays performed on readily accessible patient material may improve the interpretation of rare variants in neurodevelopmental disorders.

## 1. Introduction

*BRAT1* (BRCA1-associated ATM activator 1) encodes a multifunctional protein involved in the cellular response to DNA damage, mitochondrial homeostasis, and cellular stress regulation. *BRAT1* interacts with the ataxia-telangiectasia mutated (ATM) kinase and with components of the non-homologous end-joining machinery, and because post-mitotic neurons are particularly vulnerable to unrepaired genomic damage and mitochondrial dysfunction, disruption of *BRAT1* activity during neurodevelopment is thought to compromise neuronal survival, migration, and synaptic maturation, providing a plausible mechanistic link between *BRAT1* dysfunction and the broad range of neurological phenotypes observed in affected individuals, from severe neonatal encephalopathy to comparatively mild, later-onset cerebellar involvement.

Pathogenic biallelic *BRAT1* variants cause a broad spectrum of neurodevelopmental disorders, ranging from severe neonatal encephalopathy to comparatively mild cerebellar phenotypes that become apparent later in childhood or adolescence. The most severe form, rigidity and multifocal seizure syndrome, lethal neonatal form (RMFSL), presents in the first days of life with intractable seizures, generalized rigidity, autonomic instability, respiratory failure, and early death [1]. At the milder end of the spectrum, neurodevelopmental disorder with cerebellar atrophy with or without seizures (NEDCAS) has been described, with survival into childhood, adolescence, or adulthood and variable neurological impairment, often with an unremarkable neonatal period [2,3].

Engel and colleagues reported the largest cohort to date, comprising 97 patients, and showed that biallelic truncating variants are generally associated with the most severe, often lethal, presentations, whereas at least one variant retaining partial function tends to associate with longer survival and milder disease [4]. Carapancea and colleagues described detailed neurophysiological phenotypes in neonates with *BRAT1* encephalopathy, highlighting the diagnostic value of early EEG and evoked potential abnormalities [5].

A systematic review by Kong and colleagues, pooling PRISMA-based data from 28 studies and 50 patients, proposed unifying the fragmented nomenclature (RMFSL, NEDCAS, *BRAT1* encephalopathy) under the term *BRAT1*-related disease (BRD), and provided the first quantitative phenotype hierarchy: decreased head circumference (80%), seizures (78%), and developmental delay (76%) as high-relevance features; EEG abnormality, hypertonia/rigidity, microcephaly, and infantile death as medium-relevance features (50–75%); and apnoea, cerebellar atrophy, and hypotonia as low-relevance features (25–50%) [6]. The same review identified c.638_639insA as a mutational hotspot and showed that homozygous genotypes carry substantially higher mortality and more uniformly abnormal EEG findings than heterozygous genotypes, providing population-level support for a genotype-driven severity gradient [6]. These cohort-level findings should be interpreted cautiously in light of differences in study design, ascertainment, and depth of clinical and functional characterization.

Taken together, these observations support a broad genotype–phenotype gradient in BRAT1-related disease, with more severe presentations generally associated with biallelic truncating genotypes and milder phenotypes more frequently reported when at least one missense or hypomorphic allele is present [2,7,8], including cases of non-progressive cerebellar ataxia with comparatively preserved cognition [7,9,10].

Despite this growing clinical experience, functional validation of *BRAT1* variants remains limited, and the biological significance of most reported missense changes has not been formally established. This is an important gap, because variants of uncertain significance are frequently identified on diagnostic panels, and their classification under ACMG/AMP criteria directly influences diagnostic conclusions, prognostic counselling, and reproductive decision-making for affected families [11]. Recent guidance emphasizes integrating well-validated functional assays into variant interpretation pipelines, particularly for genes such as *BRAT1* in which the clinical spectrum is wide, and genotype alone is often insufficient to predict outcome [11,12,13].

Here, we describe the clinical, neuroimaging, genetic, structural-modelling, and BRAT1 protein-expression findings of an adolescent patient with a mild BRAT1-related phenotype carrying c.294dup in trans with the novel missense variant c.1784A>T/p.(Glu595Val).

## 2. Materials and Methods

### 2.1. Clinical Evaluation

The proband is an 18-year-old boy. Full medical and genetic assessments were performed as per clinical practice. Longitudinal neurological assessment was performed over more than fifteen years. Disease severity and functional status were quantified using the SARA scale, the FARS neurological examination and FARS-ADL subscales, the 9-Hole Peg Test, a timed mobility test (Timed-Up-and-Go, TUG), and SPATAX staging. Cognitive and behavioural assessment included the Wechsler Adult Intelligence Scale–Fourth Edition (WAIS-IV), the MT-16-19 academic skills battery, and the Kiddie Schedule for Affective Disorders and Schizophrenia (K-SADS). Family history was obtained through a structured parental interview, and available siblings were examined directly.

### 2.2. Neuroimaging and Neurophysiology

Brain and spinal MRI were performed using standard epilepsy/neurodevelopmental protocols adopted in our diagnostic assessments of children with rare diseases, and with particular attention to posterior fossa structures. Electroencephalography (EEG), brainstem auditory evoked potentials (BAEP), somatosensory evoked potentials (SSEP), and peripheral nerve conduction studies were recorded using standard clinical protocols and normative laboratory reference values, with independent review by a clinical neurophysiologist.

### 2.3. Genetic Analysis

Exome sequencing was performed on genomic DNA extracted from peripheral blood leukocytes. Genetic analysis was subsequently performed by applying an in silico ataxia gene panel to the exome sequencing dataset, allowing post-sequencing filtering of variants in 257 ataxia-related genes; the complete gene list is provided in Supplementary Table S1. Candidate variants were confirmed by Sanger sequencing in the proband, with segregation analysis extended to both parents and siblings. Variant nomenclature followed HGVS recommendations, and interpretation followed ACMG/AMP guidelines [11], applying gene- and evidence-specific specifications where available, including recommendations for functional evidence under the PS3/BS3 criterion [12,13]. Population frequency data were reviewed in large reference databases. CADD and AlphaMissense 1.0.0 were used as computational predictors for the missense variant. Structural modelling procedures are described in Section 2.5. *BRAT1* variants were annotated according to NM_152743.4, corresponding to transcript variant 2 and encoding the 821-amino-acid isoform.

### 2.4. Fibroblast Culture and Western Blot

Primary dermal fibroblasts were obtained from the patient by skin punch biopsy after informed consent, alongside fibroblasts from seven unrelated, age- and sex-matched healthy controls processed in parallel. Cells were expanded under standard conditions and harvested at comparable passage numbers. Total protein was extracted using standard lysis buffer with protease inhibitors, and equal protein amounts were loaded across samples. Proteins were separated by SDS-PAGE and transferred for immunoblotting with a primary anti-BRAT1 antibody (Abcam (Cambridge, UK), ab181855; RRID: AB_3753504) and anti-GAPDH as a loading control (manufacturer, catalogue number, dilution, and RRID for both antibodies are provided in Supplementary Table S2), followed by chemiluminescent detection. Band intensities were quantified by densitometry (ImageJ 1.54t), normalized to GAPDH, and expressed as fold change relative to the mean of the controls. Control values derive from seven distinct cell lines (biological replicates), whereas, owing to the rarity of the condition, the patient sample was loaded in two lanes from the same cell lysate (technical duplicates). Functional evidence was interpreted according to published recommendations for the PS3/BS3 criterion within the ACMG/AMP framework [13].

### 2.5. Structural Modelling

To investigate the potential structural consequences of the novel *BRAT1* missense variant p.(Glu595Val), three-dimensional structural modelling was performed using the AlphaFold-predicted structure of the human *BRAT1* protein. The wild-type protein model was visualized and analysed using PyMOL (version 3.0; Schrödinger LLC, New York, NY, USA). The p.(Glu595Val) substitution was generated in silico, and the local structural environment was compared between the wild-type and mutant proteins. Residue conservation, secondary structural context, solvent accessibility, and local intermolecular interactions were visually inspected to evaluate the potential local structural impact of the amino acid substitution.

## 3. Results

The index patient was born to non-consanguineous parents in a diamniotic dichorionic twin pregnancy. Pregnancy and delivery were uneventful. He was born at 39 weeks of gestation via spontaneous delivery, showing normal growth parameters (birth weight 2960 g, length 49 cm, head circumference 32 cm) and recording an Apgar score of 10 at both 1 and 5 min. The perinatal period was unremarkable. Early motor milestones were initially within the expected range: head control was achieved at 3 months, sitting at 5 months, and independent standing at 12 months, while autonomous walking—with a wide-based gait and frequent falls—was delayed, being achieved at approximately 2.5 years. Speech onset occurred at around 2 years of age, and was followed by persistently slurred, monotone speech with reduced prosody. The family history was unremarkable. Due to global developmental delay, the patient underwent long-term neuropsychiatric follow-up and rehabilitation until adolescence.

At the most recent evaluation, performed at the age of 18 years and 5 months, the patient presented with mild dysmorphic features, including an elongated face, short philtrum, retrognathism, and a prominent nasal root (Supplementary Figure S1). He also exhibited long fingers relative to palm length and a deep, nasal voice. Neurological examination revealed action tremor affecting the hands and tongue. No seizures were reported. We also observed gait ataxia (Supplementary Video S1), dysarthric and scanning speech, hypokinetic limb movements, impaired ocular movements with gaze-evoked horizontal nystagmus, and developmental delay. Examination further revealed brisk symmetric deep tendon reflexes with exhaustible bilateral ankle clonus, dysmetria on finger-to-nose testing, impaired single-leg stance, and a wide-based, externally rotated, propulsive gait with kyphotic posture; running was clumsy but preserved. Speech was scanning and hypophonic, though intelligible; cranial nerve and oculomotor examination were otherwise unremarkable. On interview the patient was alert, oriented, cooperative and euthymic, though behaviourally inhibited, with parent-reported mild emotional immaturity and low frustration tolerance (K-SADS). WAIS-IV testing showed a borderline, disharmonic cognitive profile (FSIQ 71 [67–77]; GAI 77 [72–83]) with comparable verbal (VCI 76) and perceptual (PRI 83) indices, relatively preserved working memory (WMI 89), and markedly deficient processing speed (PSI 58; Coding SS = 3, Symbol Search SS = 2), attributable to generalized ideomotor slowing and dysarthric, latency-prolonged verbal output. Academic skills (MT-16-19/18) showed preserved accuracy but reduced reading speed (<5th percentile) and slow, tremulous handwriting, consistent with executive-motor rather than primary learning impairment.

**Figure 1:**
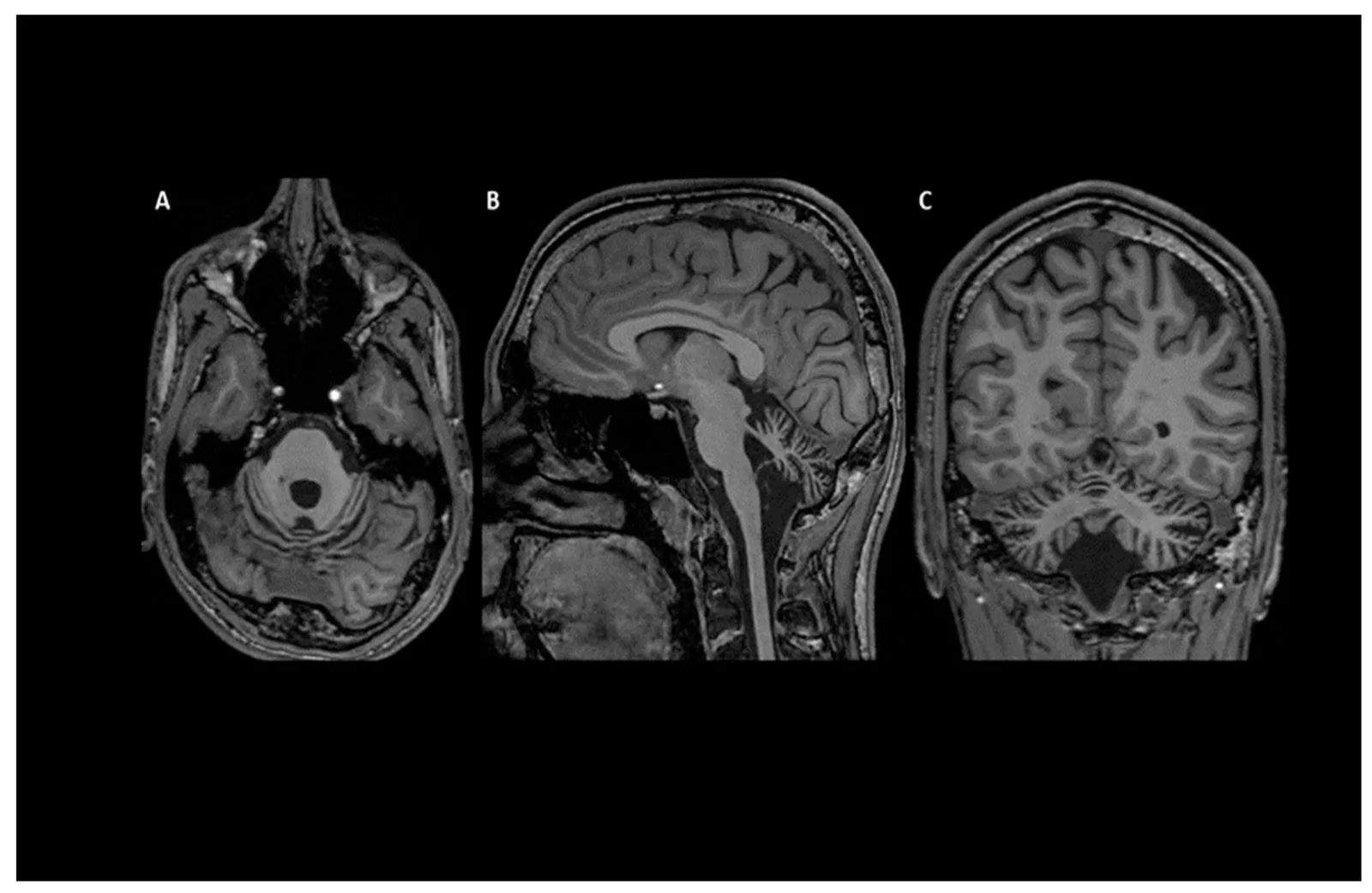
Brain 3T MRI performed at the age of 17 years. (**A**) T1-weighted axial sequence showing cerebellar hypoplasia. (**B**) T1-weighted sagittal sequence also showing cerebellar hypoplasia, particularly involving the inferior vermis, associated with a mega cisterna magna. (**C**) T2-weighted coronal sequence showing enlargement of the subtentorial cerebrospinal fluid spaces.

He scored 8.5/40 on SARA and 11/36 on FARS-ADL, with a FARS Functional Stage of 1.5. He completed the 9-Hole Peg Test in 36 s and the TUG test in 11 s. At the most recent assessment, he was attending the final year of high school and remained ambulant, indicating relatively preserved educational and functional autonomy compared with most previously reported BRAT1-related phenotypes. Brain MRI revealed marked hypoplasia of the inferior cerebellar vermis and a mega cisterna magna, with no involvement of the cerebellar hemispheres (Figure 1). The most recent MRI also suggested a possible gliotic and Wallerian component, and an incidental pineal cyst was noted on early neuroimaging.

Neurophysiological studies showed abnormalities involving the brainstem auditory pathways. An EEG performed in April 2010 demonstrated diffuse sharp transients during sleep. Follow-up EEGs performed in January 2011 and November 2025 were within normal limits, with no epileptiform discharges or photoparoxysmal responses.

Exome sequencing with in silico ataxia-panel filtering identified two heterozygous variants in *BRAT1*: a paternally inherited frameshift variant, c.294dup (p.(Leu99Thrfs\92)), and a maternally inherited missense variant, c.1784A>T (p.(Glu595Val)), not previously reported in the literature or in population and disease-specific databases at the time of analysis. Notably, c.294dup is a recurrent *BRAT1* allele, previously identified in seven families in the largest published cohort [4]. Because c.294dup-containing genotypes have been associated with both RMFSL and NEDCAS, this allele alone should not be considered predictive of a mild phenotype. Previously reported patients carrying the recurrent c.294dup variant in trans with a missense allele were therefore considered the closest available genotypic comparators to the present case [14,15]. The missense variant c.1784A>T affects a protein region conserved across vertebrate BRAT1 orthologues (Supplementary Figure S2); residue-level conservation at position 595 is most evident among mammalian orthologues. Based on the combination of rarity, in silico predictions, and the absence of definitive functional data at initial classification, the variant was classified as a variant of uncertain significance according to ACMG criteria. Segregation analysis by Sanger sequencing in the parents confirmed the two variants were in trans, consistent with compound heterozygosity in the proband. Both siblings, including the twin brother, were phenotypically normal. One sibling did not carry either of the two *BRAT1* variants, whereas the twin brother was heterozygous for the c.1784A>T/p.(Glu595Val) variant, which was inherited from the mother.

**Figure 2:**
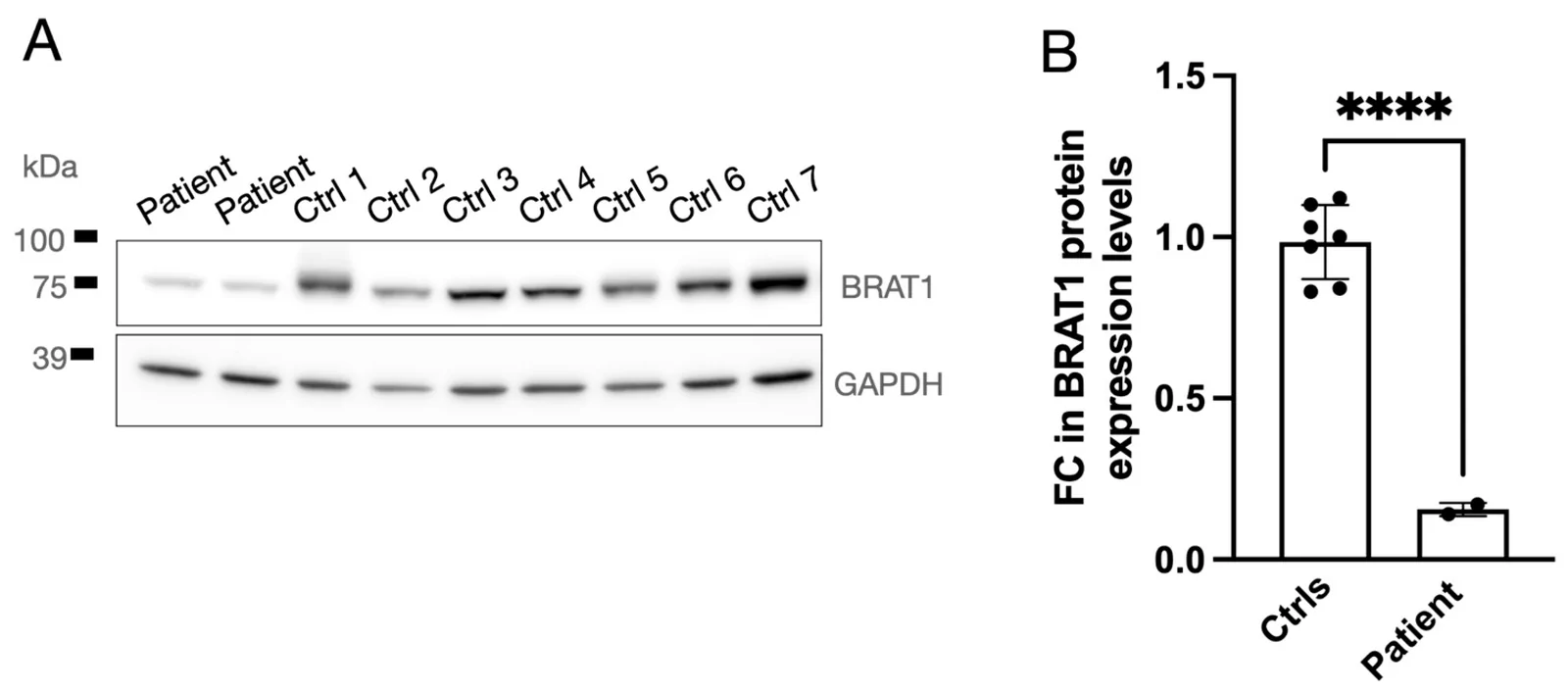
Quantitative analysis of BRAT1 protein expression in cultured skin fibroblasts. (**A**) Representative Western blot showing BRAT1 protein expression in cultured skin fibroblasts from the index case (Patient) and seven age- and sex-matched healthy control individuals (Ctrl 1–7). GAPDH was used as an internal loading control. Molecular weight markers are indicated on the left in kilodaltons (kDa). (**B**) Densitometric quantification of BRAT1 levels normalized to GAPDH are presented as fold change (FC) relative to the mean of the healthy control group. Band intensities were measured using ImageJ software (1.54t). Data points represent individual values for n = 7 biological control replicates and n = 2 technical duplicates of the same cell lysate loaded in separate lanes for the patient. Bars represent the mean ± SD. For descriptive purposes only, given that the patient values are technical duplicates of a single lysate, statistical significance was calculated using an unpaired Welch’s *t*-test (**** *p* < 0.0001).

Western blot analysis of patient-derived fibroblasts demonstrated a marked reduction in BRAT1 protein levels compared with controls, with densitometric quantification indicating an approximately 80–85% decrease (Figure 2). Residual BRAT1 protein was detectable in all patient lanes, indicating reduced but retained protein expression in this patient-derived cellular model.

### Structural Modelling and Mutational Landscape

Structural modelling further informed the interpretation of the missense variant. Human *BRAT1* is an 821-residue, predominantly α-helical protein that lacks any recognisable catalytic domain [16]. Its N-terminal region folds into a CIDE-N-like domain, homologous to the domain of cell-death-inducing DFF45-like effector proteins, while its C-terminal third contains two tandem HEAT repeats (approximately residues 495–531 and 547–574), paired antiparallel α-helical units that stack into an elongated, superhelical solenoid characteristic of HEAT/ARM-repeat scaffolding proteins [16]. This architecture, devoid of enzymatic motifs, is consistent with *BRAT1* acting as a protein–protein interaction scaffold rather than an enzyme: it couples ATM activation to the non-homologous end-joining machinery during the DNA damage response, contributes to mTOR stabilisation [17], and, as recently shown, directly binds and stabilises the INTS9–INTS11 catalytic heterodimer of the Integrator complex, an interaction required for correct 3′-end RNA processing and for removal of the transcriptional repressor REST from chromatin, a step required for activation of neuronal differentiation genes [18,19].

**Figure 3:**
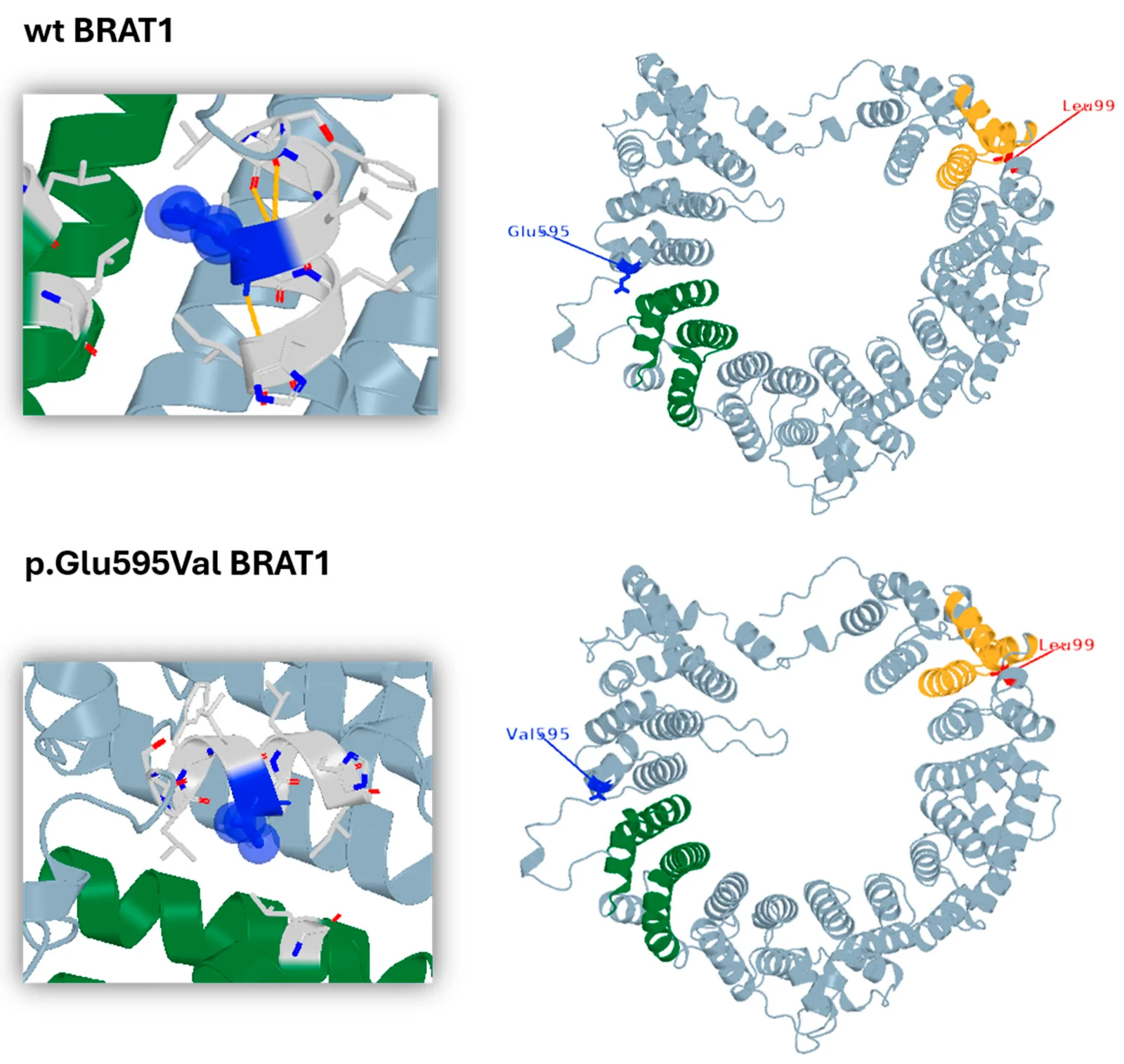
AlphaFold-based structural model of human BRAT1 generated with PyMOL v3.0. The upper panels show the wild-type BRAT1 structure, whereas the lower panels show the p.Glu595Val mutant model; both structures are explicitly labelled in the figure. Residue 595 is shown in blue. The green region highlights the adjacent α-helical segment used to orient the local structural environment around residue 595. Leu99 is indicated in red, and the adjacent yellow region is shown to orient its position in the global BRAT1 model. The insets magnify the local environment of residue 595 in the wild-type and mutant models.

The AlphaFold-based model of wild-type *BRAT1* reproduced this overall architecture, showing an elongated arrangement of stacked α-helices with comparatively few loop or β-strand elements (Figure 3). Residue Glu595 is located within the C-terminal HEAT-repeat module, adjacent to the second HEAT repeat, where it packs against the preceding helical turn. In the wild-type model, the negatively charged glutamate side chain projects toward this neighbouring helix, consistent with a contribution to local helix–helix packing through polar or electrostatic contacts. Substitution with valine, a small, uncharged, branched aliphatic residue, removes this side chain and may alter these local contacts without introducing evident steric clashes or major backbone rearrangement, raising the possibility of a local structural effect on the HEAT-repeat module without providing evidence for global protein misfolding. This structural prediction is consistent with the Western blot finding of markedly reduced but retained BRAT1 protein expression and provides a hypothesis-generating structural context for the possible residual-function effect suggested by the protein-expression data.

**Figure 4:**
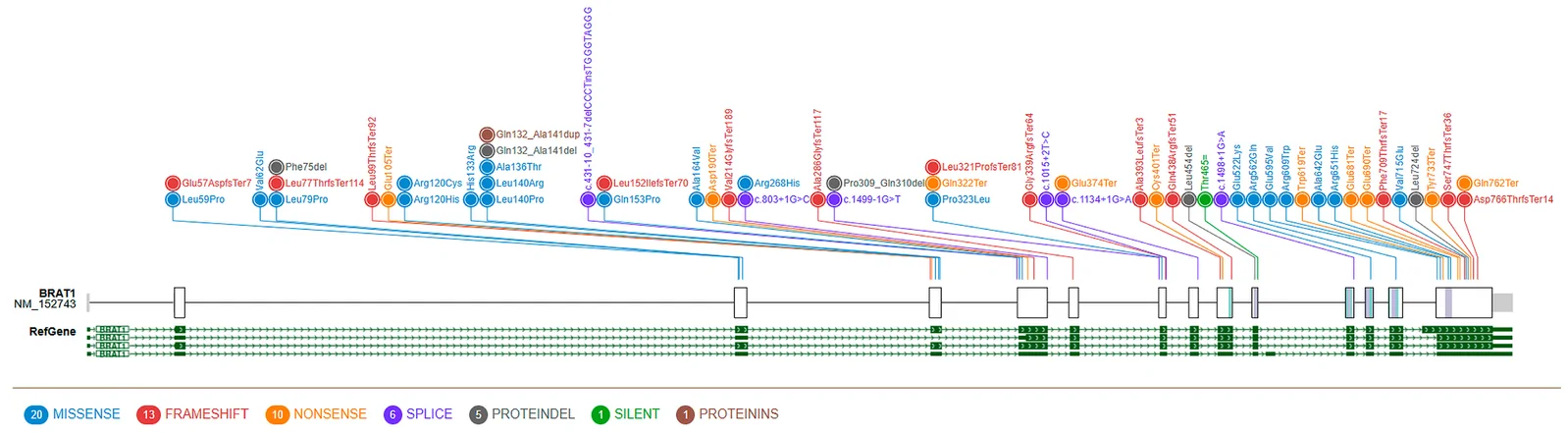
Schematic representation of variants mapped along the BRAT1 gene and the encoded protein. The figure includes 56 previously reported BRAT1 variants together with the two variants identified in the present patient. Variants are shown as colour-coded nodes and linked to their corresponding positions across the BRAT1 transcript isoforms shown at the bottom in green. These transcript tracks are provided for genomic context; variant nomenclature and amino-acid numbering refer to NM_152743.4, corresponding to the 821-amino-acid BRAT1 isoform used throughout the manuscript. Variant classes are indicated as follows: missense (blue), frameshift (red), nonsense (orange), splice-site (purple), synonymous/silent (green), and protein-level annotations (brown). Variants identified in the present study are boxed and annotated alongside those previously reported in the literature. The N-terminal CIDE-N-like domain and the C-terminal tandem HEAT-repeat module are annotated on the protein schematic to facilitate structural interpretation.

Mapping of previously reported BRAT1 variants onto the gene and protein sequence (Figure 4) showed that truncating and missense variants are distributed across different regions of the protein. Among previously reported missense variants, p.(Val62Glu), associated with non-progressive cerebellar ataxia, maps to the N-terminal CIDE-N-like domain [9], whereas the novel p.(Glu595Val) variant identified in the present patient maps to the C-terminal HEAT-repeat region. Recent case reports have further expanded the mutational landscape of BRAT1, including a missense variant associated with NEDCAS and seizures [20], a deep intronic splicing variant associated with a cerebellar hypoplasia syndrome [21], and compound heterozygous variants, one of them a synonymous splicing variant, in a patient with RMFSL [22]. Together with the Western blot findings, these data are compatible with, but do not establish, a residual-function effect of p.(Glu595Val) in trans with the recurrent truncating variant and should therefore be regarded as supportive rather than definitive functional evidence.

## 4. Discussion

This study describes an adolescent patient with a mild *BRAT1*-related phenotype, expanding the known clinical and molecular spectrum of this disorder. The combination of early-onset global developmental delay, borderline cognitive functioning, clinically stable cerebellar ataxia, dysarthria, action tremor, and oculomotor abnormalities, without epilepsy and with relatively preserved functional autonomy into late adolescence, places this patient at the mild end of the reported BRAT1-related presentations.

Compared with previously published cohorts, our patient represents a notable outlier. In the largest available cohort (97 patients), truncating variants were broadly associated with more severe disease [4], while a systematic review of 50 patients reported seizures in 78% of cases and overall mortality of approximately 62% [6]. In contrast, our patient has remained entirely seizure-free through more than fifteen years of follow-up, reaching late adolescence with relatively preserved motor and educational functioning, despite a borderline cognitive profile, while remaining independently ambulant. This divergence reinforces the concept, proposed by others describing similarly mild presentations [7,8,9], that *BRAT1*-related disorders form a continuous phenotypic spectrum rather than discrete clinical entities.

Our functional findings are consistent with previously reported genotype–phenotype correlations, in which biallelic truncating variants are generally associated with more severe disease, whereas genotypes carrying at least one partially functional allele may be associated with attenuated phenotypes [2,8]. Western blot demonstrated clearly detectable, although markedly reduced, BRAT1 protein in patient-derived fibroblasts; however, these data do not by themselves establish p.(Glu595Val) as a hypomorphic allele or demonstrate preservation of BRAT1 function. Because the c.294dup allele is expected to cause a frameshift with probable nonsense-mediated decay, the residual protein most likely derives from the p.(Glu595Val) allele, and the low level observed (about 15–20% of control) may therefore also reflect reduced stability of the missense protein; RNA expression was not assessed, and analysis of the heterozygous twin brother and of the parents could help to discriminate these possibilities. Markedly reduced BRAT1 protein levels have also been reported in cells from siblings homozygous for p.(Val62Glu) who have a non-progressive cerebellar ataxia [9], indicating that low protein levels can coexist with a mild phenotype. This interpretation is consistent with Engel et al., who reported RMFSL predominantly in association with biallelic null genotypes and NEDCAS more frequently in genotypes carrying at least one missense variant [4]. Accordingly, the recurrent c.294dup allele should be interpreted in the context of the variant present in trans and residual BRAT1 expression rather than as an independent predictor of clinical severity, consistent with the broader cohort-level severity gradient reported by Kong et al. [6]. A direct comparison with the two previously reported patients carrying c.294dup in trans with a missense allele further illustrates the phenotypic variability associated with this recurrent variant and highlights the comparatively attenuated clinical course observed in the present case (Table 1). Notably, the two comparators carry missense variants (p.Arg609Trp and p.Ala642Glu) that also lie in the C-terminal region and were nonetheless associated with epilepsy and more severe early courses, so C-terminal location alone does not explain the milder phenotype of the present case.

The structural modelling data (Figure 3 and Figure 4) provide a molecular framework that complements this genotype–phenotype model. Beyond its long-recognised role in ATM activation and non-homologous end-joining, *BRAT1* has recently been shown to bind and stabilise the INTS9–INTS11 catalytic heterodimer of the Integrator complex through its C-terminal region, an interaction required for correct 3′-end processing of small nuclear and other non-coding RNAs and removal of the transcriptional repressor REST from chromatin. This is a step required for REST-dependent activation of neuronal differentiation genes [18,19]. Because p.(Glu595Val) is located within the C-terminal region of BRAT1, the variant could in principle interfere with Integrator binding; conversely, the limited local structural impact predicted by modelling and the residual protein expression observed in our patient might indicate that these interactions are at least partly retained, which could plausibly be relevant to the attenuated clinical phenotype. This remains speculative (see above). In this context, a partial preservation of BRAT1 interactions involving the Integrator complex and mTOR pathway may represent one possible biological explanation. However, this interpretation remains hypothesis-generating, since the present study did not directly assess these pathway-specific functions. Systematic structural annotation of future *BRAT1* variants, combined with a more standardised measure of residual protein function, could also help to refine the interpretation of the contribution of individual protein domains to BRAT1-related phenotypic variability.

Neuroimaging findings further support this interpretation. The presence of isolated cerebellar vermian hypoplasia, together with prolonged clinical stability, favours a predominantly developmental mechanism rather than an actively progressive neurodegenerative process, consistent with the proposed role of *BRAT1* during early neurodevelopment. Although the most recent MRI suggested a possible gliotic and Wallerian component, the clinical stability over prolonged follow-up argues against ongoing structural degeneration, although only one MRI (age 17 years) is reported here. Continued longitudinal imaging will nevertheless be important to clarify whether these findings represent static developmental sequelae or very slowly evolving secondary changes.

This case also highlights the importance of integrating functional evidence into the interpretation of rare missense variants, particularly for genes such as *BRAT1*, in which genotype alone is often insufficient to predict disease severity. Well-validated functional assays can provide moderate to strong evidence toward pathogenicity under ACMG/AMP recommendations when appropriately designed and interpreted [11,12,13]. However, the assay used here has not been validated with known pathogenic and benign control variants, so it cannot be used to apply PS3 above supporting strength. Previous functional studies in *BRAT1*-related disease have relied mainly on indirect mitochondrial biomarkers, including reduced cytochrome c oxidase staining, decreased pyruvate dehydrogenase activity, or impaired respiratory chain complex IV activity, none of which has proven consistently reproducible across affected individuals, and no disease-specific biomarker has yet been established [6]. By contrast, direct quantification of *BRAT1* protein represents a more proximal and gene-specific measure of the molecular consequence of a variant. Importantly, this approach can be performed using readily accessible patient-derived fibroblasts obtained from minimally invasive skin biopsy, without requiring neuronal tissue or complex experimental models. In the present case, demonstration of reduced but detectable BRAT1 protein expression was compatible with a residual-function model, without establishing it, and added functional information to the molecular interpretation of the novel missense variant.

The present case also emphasizes that the milder end of the *BRAT1* spectrum may remain underrecognized. In the absence of neonatal encephalopathy or epilepsy, affected individuals may instead receive nonspecific diagnoses such as congenital ataxia or static cerebellar syndrome, delaying molecular diagnosis for many years. Recognition that *BRAT1* pathogenic variants may present with non-progressive cerebellar ataxia, developmental delay of variable degree, and relatively preserved functional independence broadens the clinical indications for genetic testing. Earlier diagnosis may facilitate more accurate prognostic counselling, avoid unnecessary investigations, and improve long-term multidisciplinary management while refining genotype–phenotype correlations through identification of additional mild cases.

We acknowledge multiple limitations in our study. Functional assessment was restricted to BRAT1 protein quantification in patient-derived fibroblasts. Although reduced but detectable BRAT1 expression was observed, patient values derive from technical duplicates of a single cell lysate (no independent biological replicates were available), and protein stability was not directly tested by cycloheximide-chase assay; therefore, the reduced BRAT1 signal cannot be specifically attributed to accelerated protein degradation. In addition, we did not assess BRAT1 subcellular localization, ATM-dependent signalling, DNA-damage response, mitochondrial function, Integrator-complex activity, or neuronal-specific phenotypes. Accordingly, the Western blot data should be interpreted as supportive evidence of reduced but retained BRAT1 expression, compatible with but not sufficient to establish a hypomorphic effect, rather than as definitive proof of the underlying molecular mechanism. Because this is a single-patient report, the genotype–phenotype interpretation and the specific contribution of p.(Glu595Val) require confirmation in additional patients and complementary functional models. In addition, exome data were analysed through an ataxia-gene filter, so a contribution of variants in other genes to the broader neurodevelopmental phenotype cannot be entirely excluded. Finally, the incidental pineal cyst observed on early neuroimaging was not specifically reassessed, and continued clinical and neuroradiological follow-up will be useful to document long-term stability and clarify the significance of subtle MRI changes over time. Structural modelling should also be interpreted as qualitative and hypothesis-generating. The analysis was based on AlphaFold-derived modelling and PyMOL visual inspection of the local environment of p.Glu595Val, without quantitative stability calculations or biochemical validation of the predicted local contacts. Therefore, these data support only a possible local structural effect and cannot establish the molecular consequence of the variant.

## 5. Conclusions

This study expands the clinical and molecular spectrum of *BRAT1*-related disorders by describing a novel p.(Glu595Val) variant in trans with the recurrent c.294dup, associated with reduced but detectable protein expression in patient-derived fibroblasts and a mild neurodevelopmental phenotype in a patient with relatively preserved functional autonomy and no history of epilepsy. Whether the residual-function model suggested by this single case provides a useful reference point for the interpretation of comparable *BRAT1* genotypes remains a hypothesis-generating question. Confirmation through independent cases and more robust functional and structural validation will be necessary.

## Figures and Tables

**Table 1:** Clinical comparison of patients carrying the recurrent BRAT1 c.294dup variant in trans with a missense allele.

Case	BRAT1 Genotype	Epilepsy	Developmental Outcome	Neuroimaging	Follow-Up
**Mundy et al.** [15]	c.294dup/c.1925C>A; p.Leu99Thrfs*92/p.Ala642Glu	Present	Neurodevelopmental impairment; early RMFSL-like presentation	Decreased myelination, thin corpus callosum, cerebellar hypoplasia, right temporal encephalomalacia	Alive at 6 years
**Hanes et al.** [14]	c.294dup/c.1825C>T; p.Leu99Thrfs*92/p.Arg609Trp	Present	Global developmental impairment with continued acquisition of developmental skills	Cerebellar and brainstem atrophy with dysmyelination	Alive at 3 years 8 months
**Present case**	c.294dup/c.1784A>T; p.Leu99Thrfs*92/p.Glu595Val	Absent	Childhood global developmental delay; borderline cognitive profile; attending the final year of high school	Inferior vermian hypoplasia with mega cisterna magna; no cerebellar hemispheric involvement	Alive at 18 years 5 months

## Data Availability

No new data were created or analyzed in this study. Data sharing is not applicable to this article.

## Supplementary Materials

The following supporting information can be downloaded at: https://www.mdpi.com/article/10.3390/medsci14060641/s1, Supplementary Figure S1. Facial features. Frontal and lateral views show a long face, short philtrum, retrognathism and a prominent nasal root. Supplementary Figure S2. Evolutionary conservation of the BRAT1 region surrounding human Glu595. Multiple-sequence alignment of BRAT1 orthologues from zebrafish (*Danio rerio*), frog (*Xenopus tropicalis*), chicken (*Gallus gallus*), mouse (*Mus musculus*), human (*Homo sapiens*), and dog (*Canis lupus familiaris*), focused on the amino-acid region surrounding human Glu595. The boxed column indicates the position corresponding to human Glu595, the residue affected by the p.Glu595Val variant, and shows the aligned residues at the equivalent position in other vertebrate orthologues. The alignment supports conservation of the local BRAT1 sequence context around Glu595 across vertebrates, while residue-level conservation at the corresponding position is most evident among mammalian sequences. Amino-acid numbering refers to NM_152743.4, corresponding to the 821-amino-acid BRAT1 isoform used throughout the manuscript. Supplementary Table S1. Genes included in the in- silico ataxia gene panel. Gene symbols included in the in-silico ataxia gene panel used for post-sequencing variant filtering. Supplementary Video. Neurological examination of the index case.

## Abbreviations

ACMG	American College of Medical Genetics and Genomics
AMP	Association for Molecular Pathology
ATM	Ataxia-telangiectasia mutated
BAEP	Brainstem auditory evoked potentials
BRAT1	BRCA1-associated ATM activator 1
BRD	BRAT1-related disease
FARS-ADL	Friedreich Ataxia Rating Scale–Activities of Daily Living
GAPDH	Glyceraldehyde-3-phosphate dehydrogenase
HGVS	Human Genome Variation Society
MRI	Magnetic resonance imaging
NEDCAS	Neurodevelopmental disorder with cerebellar atrophy with or without seizures
RMFSL	Rigidity and multifocal seizure syndrome, lethal neonatal form
SARA	Scale for the Assessment and Rating of Ataxia
SDS-PAGE	Sodium dodecyl sulfate–polyacrylamide gel electrophoresis
SPATAX	Spinocerebellar ataxia/spastic ataxia staging system
SSEP	Somatosensory evoked potentials
TUG	Timed Up-and-Go
