# Supplementary material for: A Mild Neurodevelopmental Phenotype Associated with a Novel BRAT1 Variant: Expanding Genotype–Phenotype Correlations
Source: Med Sci (Basel). 2026 Oct 6;14(6):641. doi: 10.3390/medsci14060641 (PMC13706764; doi:10.3390/medsci14060641)
Supplement: Supplementary file 1 [file medsci-14-00641-s001.zip › BRAT1_supplementary_materials_09.09.26.pdf]

Supplementary Materials

Supplementary Table S1. Genes included in the in- silico ataxia gene panel.
Gene symbols included in the in-silico ataxia gene panel used for post-sequencing variant filtering.

Gene symbol	Gene symbol	Gene symbol	Gene symbol	Gene symbol
AARS2	*ABCB7*	*ABCD1*	*ABHD12*	*ACO2*
ADCK3	*ADGRG1*	*AFG3L2*	*AHDC1*	*AHI1*
ALDH5A1	*ALG3*	*ALG6*	*AMACR*	*AMPD2*
ANO10	*APOB*	*APTX*	*ARL13B*	*ARSA*
ATCAY	*ATG5*	*ATL1*	*ATM*	*ATP13A2*
ATP1A2	*ATP1A3*	*ATP2B3*	*ATP7B*	*ATP8A2*
BEAN1	*BRAT1*	*BRF1*	*C10orf2*	*C12orf65*
C19orf12	*C5orf42*	*C9orf72*	*CA8*	*CACNA1A*
CACNA1G	*CACNB4*	*CAMTA1*	*CASK*	*CC2D2A*
CCDC88C	*CD40LG*	*CDK5*	*CEP104*	*CEP290*
CEP41	*CHMP1A*	*CLCN2*	*CLN5*	*CLN6*
CLN8	*CLP1*	*COA7*	*COQ2*	*COQ4*
COQ9	*COX20*	*CP*	*CSPP1*	*CSTB*
CTBP1	*CTSD*	*CWF19L1*	*CYP27A1*	*CYP7B1*
DAB1	*DARS*	*DARS2*	*DDHD2*	*DKC1*
DNAJC19	*DNAJC3*	*DNMT1*	*EEF2*	*EIF2B1*
EIF2B2	*EIF2B3*	*EIF2B4*	*EIF2B5*	*ELOVL4*
ELOVL5	*ERCC4*	*ERCC8*	*EXOSC3*	*EXOSC8*
FA2H	*FARS2*	*FASTKD2*	*FAT2*	*FGF14*
FLVCR1	*FMR1*	*FOLR1*	*FXN*	*GALC*
GAN	*GBA2*	*GBE1*	*GFAP*	*GJB1*
GJC2	*GLB1*	*GOSR2*	*GRID2*	*GRM1*
HARS	*HARS2*	*HEXA*	*HEXB*	*HIBCH*
HSD17B4	*INPP5E*	*ITPR1*	*KCNA1*	*KCNA2*
KCNC1	*KCNC3*	*KCND3*	*KCNJ10*	*KCNMA1*
KCTD7	*KIF1A*	*KIF1C*	*KIF7*	*LAMA1*
LMNB2	*LYST*	*MARS2*	*MED17*	*MFN2*
MFSD8	*MKS1*	*MMACHC*	*MME*	*MRE11A*
MTPAP	*MTTP*	*MVK*	*NAGLU*	*NDUFS1*
NDUFS7	*NEU1*	*NOP56*	*NPC1*	*NPC2*
NPHP1	*OFD1*	*OPA1*	*OPA3*	*OPHN1*
PAX6	*PCLO*	*PCNA*	*PDE6D*	*PDHA1*
PDSS1	*PDSS2*	*PDYN*	*PEX10*	*PEX16*
PEX2	*PEX6*	*PEX7*	*PHYH*	*PIK3R5*
PLA2G6	*PLD3*	*PLP1*	*PMM2*	*PMPCA*
PNKP	*PNPLA6*	*POLG*	*POLR3A*	*POLR3B*
PPT1	*PRICKLE1*	*PRKCG*	*PRNP*	*PRPS1*

Gene symbol	Gene symbol	Gene symbol	Gene symbol	Gene symbol
PRRT2	*PSAP*	*PSEN1*	*PTF1A*	*PTRH2*
PUM1	*QARS*	*RAB3GAP1*	*RARS*	*RARS2*
RELN	*RNF170*	*RNF216*	*RPGRIP1L*	*RUBCN*
SACS	*SAMD9L*	*SARS*	*SCN1A*	*SCN2A*
SCN8A	*SCYL1*	*SEPSECS*	*SETX*	*SIL1*
SLC17A5	*SLC1A3*	*SLC25A46*	*SLC2A1*	*SLC33A1*
SLC35A2	*SLC52A2*	*SLC6A19*	*SLC9A1*	*SLC9A6*
SMPD1	*SNAP25*	*SNX14*	*SPAST*	*SPG11*
SPG7	*SPTAN1*	*SPTBN2*	*SRD5A3*	*STS*
STUB1	*STXBP1*	*SURF1*	*SYNE1*	*SYNE2*
SYT14	*TBC1D23*	*TCTN1*	*TCTN2*	*TCTN3*
TDP1	*TDP2*	*TGM6*	*TMEM138*	*TMEM216*
TMEM231	*TMEM237*	*TMEM240*	*TMEM67*	*TOE1*
TOP1	*TPP1*	*TRNT1*	*TRPC3*	*TSEN15*
TSEN2	*TSEN34*	*TSEN54*	*TTBK2*	*TTC19*
TTC21B	*TTPA*	*TUBB3*	*TUBB4A*	*UBA5*
UBR4	*UCHL1*	*VAMP1*	*VARS2*	*VLDLR*
VPS53	*VRK1*	*VWA3B*	*WDR73*	*WDR81*
WFS1	*WWOX*	*XPA*	*XRCC1*	*XRCC4*
ZFYVE26	*ZFYVE27*	*ZNF423*		

Supplementary Figure S2. Evolutionary conservation of the BRAT1 region surrounding human Glu595. Multiple-sequence alignment of BRAT1 orthologues from zebrafish (*Danio rerio*), *Xenopus tropicalis*, chicken (*Gallus gallus*), mouse (*Mus musculus*), human (*Homo sapiens*), and dog (*Canis lupus familiaris*), focused on the amino-acid region surrounding human Glu595. The boxed column indicates the position corresponding to human Glu595, the residue affected by the p.Glu595Val variant, and shows the aligned residues at the equivalent position in other vertebrate orthologues. The alignment supports conservation of the local BRAT1 sequence context around Glu595 across vertebrates, while residue-level conservation at the corresponding position is most evident among mammalian sequences. Amino-acid numbering refers to NM_152743.4, corresponding to the 821-amino-acid BRAT1 isoform used throughout the manuscript.

Human Glu595 position

```
sp|Q1RLU1|BRAT1_DANRE              I---NLLEEALAQMMIILSQDTEGFPRRAVVKTFTSWLKGSNPITAL--DSSLSSVLSLG    629
tr|F6VIU7|F6VIU7_XENTR             NWLPINTEDIASHLLDILSQDTESFPRRAAVKVFTDWLKQGHMQHFKDPKQMLSKILEVT    641
tr|A0A8V0ZBQ7|A0A8V0ZBQ7_CHICK     AGNQYNKEKTVAKLQEILSTDPEGFPRRAVMNVFTMWLKESCTGQLEETEQFVSTVVQTT    652
sp|Q8C3R1|BRAT1_MOUSE              NSQ--AQQGLLMDLMHILSTDSEGFPRRAVLRVFTDWLRDGHADVVRDTEWFVATVLQAV    643
sp|Q6PJG6|BRAT1_HUMAN              HAE--ARQSLFLELLHILSVDSEGFPRRAVMQVFTEWLRDGHADAAQDTEQFVATVLQAA    642
tr|A0A8I3S1P3|A0A8I3S1P3_CANLF     HPG--GQQSLLVELLHILSTDSEGFPRRAVMQVFTQWLRDHHADVAGDTEQFMARVLQVA    643
```
