# Supplementary material for: A Mild Neurodevelopmental Phenotype Associated with a Novel BRAT1 Variant: Expanding Genotype–Phenotype Correlations
Source: Med Sci (Basel). 2026 Oct 6;14(6):641. doi: 10.3390/medsci14060641 (PMC13706764; doi:10.3390/medsci14060641)
Supplement: Supplementary file 1 [file medsci-14-00641-s001.zip › medsci-4584166 Full Length BRAT1.pdf]

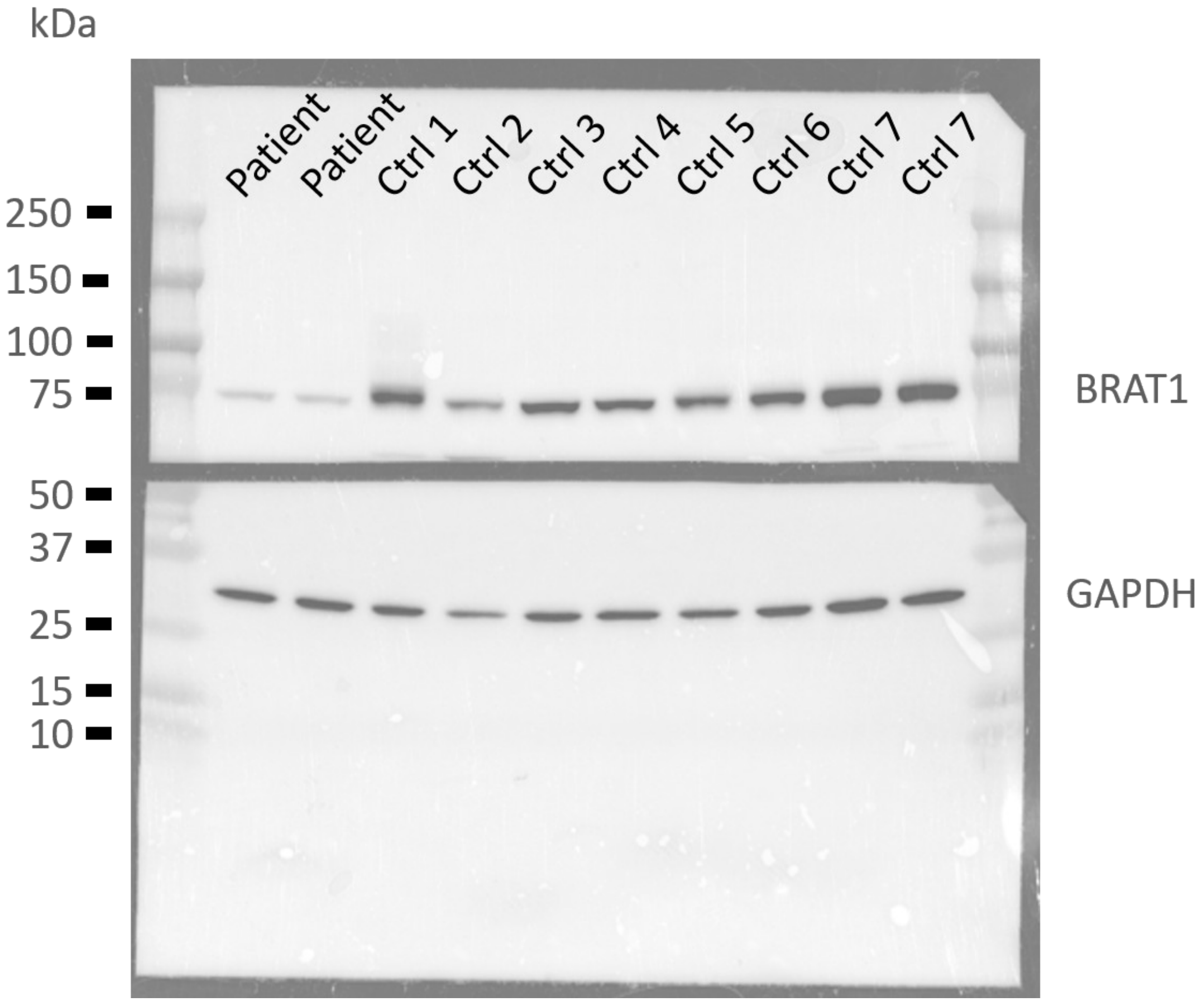
kDa
250
150
100
75
50
37
25
15
10
Patient
Patient
Ctrl 1
Ctrl 2
Ctrl 3
Ctrl 4
Ctrl 5
Ctrl 6
Ctrl 7
Ctrl 7
BRAT1
GAPDH
