# Supplementary material for: A Mild Neurodevelopmental Phenotype Associated with a Novel BRAT1 Variant: Expanding Genotype–Phenotype Correlations
Source: Med Sci (Basel). 2026 Oct 6;14(6):641. doi: 10.3390/medsci14060641 (PMC13706764; doi:10.3390/medsci14060641)
Supplement: Supplementary file 1 [file medsci-14-00641-s001.zip › medsci-4584166- Uncropped WB images.pdf]

BRAT1_1a1000_11-03-2026(Chemiluminescence).raw

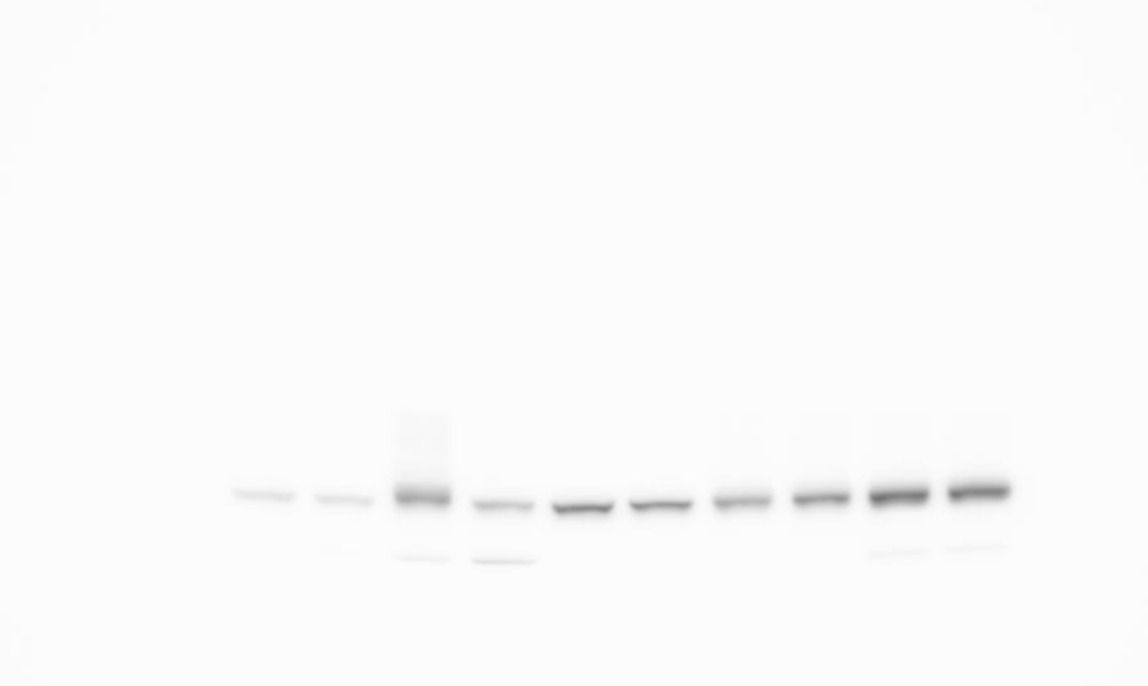

_BRAT1_1a1000_11-03-2026_MARKER(Colorimetric) uncropped.raw

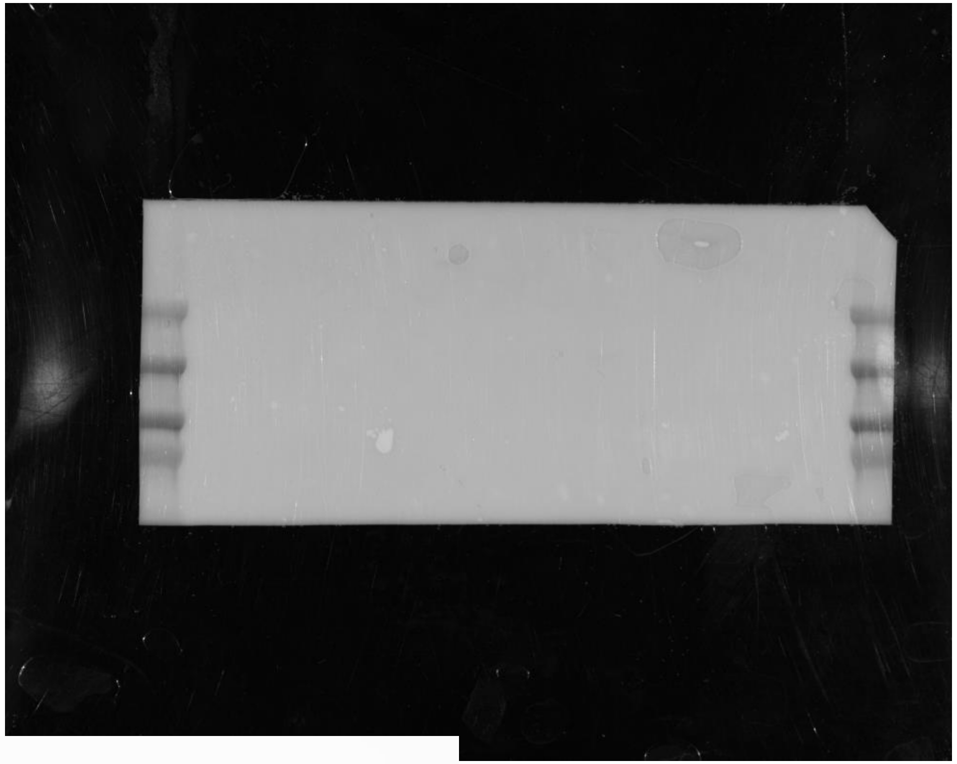

Marker_GAPDHxBRAT1_ 11-03-2026 uncropped raw

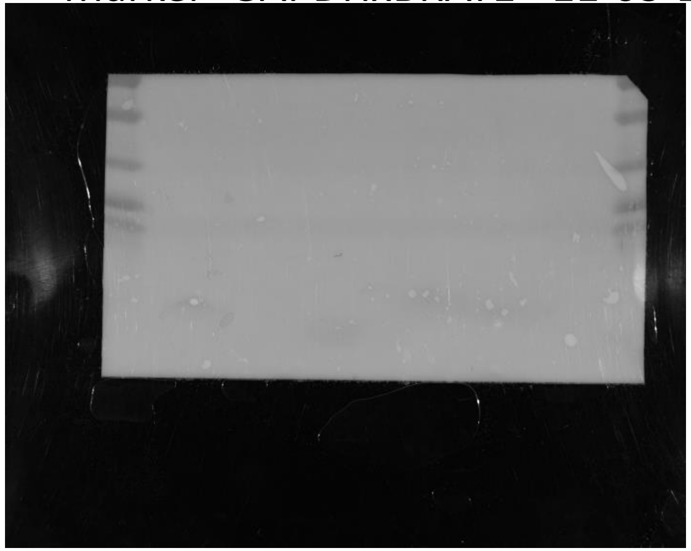

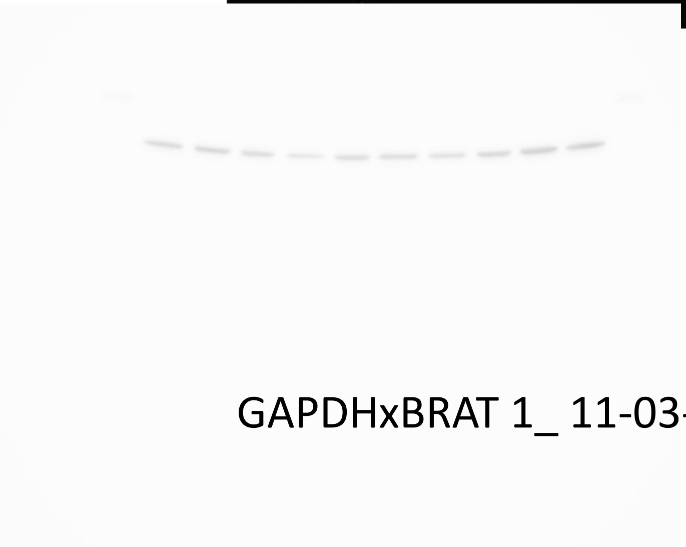

GAPDHxBRAT 1_ 11-03-2026(Chemiluminescence)
